# High chymotrypsin-like activity in the plasma of patients with newly diagnosed multiple myeloma treated with bortezomib is predictive of a better response and longer PFS

**DOI:** 10.1007/s00277-018-3393-7

**Published:** 2018-06-26

**Authors:** Wioletta Romaniuk, Lukasz Bolkun, Joanna Kalita, Marzenna Galar, Malgorzata Bernatowicz, Halina Ostrowska, Janusz Kloczko

**Affiliations:** 1 Department of Haematology, Medical University of Bialystok, Bialystok, Poland; 2 Department of Biology, Medical University of Bialystok, Bialystok, Poland

**Keywords:** Proteasome, Multiple myeloma, Chymotrypsin-like activity, Bortezomib

## Abstract

Proteasome inhibitors (PIs) such as bortezomib constitute an important part of the modern standard therapy for multiple myeloma (MM). In this study, we set out to assess whether proteasome concentration and chymotrypsin-like (ChT-L) activity could serve as potential biomarkers defining the likelihood of response to treatment with bortezomib, in order to identify patients who are more likely to respond to treatment with PI. We analysed proteasome concentration and ChT-L activity in the plasma of 78 patients with newly diagnosed MM during treatment with or without proteasome inhibitors. Values of all the studied parameters in the group of responders decreased sharply from the initial levels already after the third cycle of chemotherapy and remained significantly lower until the end of treatment. On the other hand, in the group of non-responders, there was an increase in the measured proteasome parameters already after the third cycle, and they remained high during the next cycles of therapy. We also showed that high baseline proteasome ChT-L activity values might prognosticate longer progression-free survival (PFS) in patients treated with PI. Our findings demonstrate that measuring plasma proteasome ChT-L activity can be used as a powerful biomarker for predicting clinical response to treatment and PFS in patients with newly diagnosed MM.

## Introduction

Multiple myeloma (MM) is a malignant disease characterised by proliferation of clonal plasm cells in the bone marrow, production of monoclonal proteins in the blood and/or urine and organ dysfunction [1, 2]. Although there has been a growing interest in MM treatment over the last few years, the disease still remains incurable. Introduction of high-dose chemotherapy with autologous stem cell transplantation (HDT/autoSCT), followed by new drugs, significantly improved the prognosis of MM patients, increasing both their response rates and survival parameters [3]. The release of bortezomib in 2003 by the US Food and Drug Administration (FDA) was a major breakthrough in the treatment of MM. This first-generation selective reversible proteasome inhibitor (PI) constituted the main therapeutic strategy for myeloma patients [4, 5]. Moreover, application of bortezomib in combination with immunomodulatory agents in newly diagnosed MM patients has been shown to produce impressive response rates. Nevertheless, there is still no known biomarker which would accurately predict the response and clinical outcomes.

In this context, recent studies have considered the assessment of proteasomal system to be useful in diagnosis, prognosis and monitoring of anticancer treatment of patients with haematological malignancies and other diseases [6, 7]. Proteasomes are important nonlysosomal multisubunit proteolytic complexes, whose elementary function consists in the degradation of damaged and short half-life proteins. Therefore, proteasomes play a very important role in preserving cell homeostasis and controlling various selected processes in the cells, e.g. cell cycle control (cyclins, cdk inhibitors), oncogenic transformation (N-myc, c-jun, c-fos), tumour suppression (p53), promotion of apoptosis (Bax, Bid), inhibition of apoptosis (Bcl-2, c-IAP1, XIAP) and regulation of transcription factors (NF-κB) [8]. Proteasome inhibition leads to accumulation of damaged proteins, which in turn results in caspase activation and cell death. This process is used in cancer treatment, especially in the multiple myeloma therapy [4].

Proteasomes are present in the cytoplasm and nucleus of most eukaryotic cells. Within the cells, they exist both in a free latent form and an active form known as proteasome 26S [6]. Free proteasomes, designated as circulating proteasomes, have also been detected in the human plasma, which suggests that they are its constitutive components. In healthy individuals, free proteasomes are not present in large quantities, and the values decrease or increase in the presence of diseases. Thus, their level may reflect the health status of a given person [9]. Several recent publications have suggested that measuring proteasome concentration in the plasma with the use of an enzyme-linked immunosorbent assay (ELISA) may constitute a new method of diagnosis [10–12]. The ELISA method determines the total concentration of proteasomes (20S proteasome, free subunits and fragment subunit), whereas the ChT-L activity assay is used exclusively for the detection of enzymatically active undamaged proteasome 20S.

The 26S proteasome is comprised of a core, 20S proteasome and two cap-19S regulatory complex. The 20S proteasome is a cylinder formed by four rings, each containing seven different subunits. Only three inner β-subunits, however, contain the protease active sites: β5-subunit demonstrates a chymotrypsin-like activity (ChT-L), β2-subunit a trypsin-like activity (T-L) and β1-subunit a caspase-like activity (Cas-L) (peptidyl-glutamyl peptide-hydrolazing-like (PGPH-like)) [13]. PIs approved by FDA, selectively and reversibly (bortezomib- VELCADE®, ixazomib- NINLARO®), as well as irreversibly (carfilzomib-KYPROLIS®), inhibit the proteasome ChT-L activity, responsible for limiting the intracellular degradation of proteins, especially the ones that determine tumour growth and survival, and to a lesser extent the caspase-like and trypsin-like activities [14, 15]. On the other hand, inhibition of the ChT-L activity stops cell cycle progression and induces apoptosis in the neoplastic cells of haematological malignancies and solid tumours [10]. Earlier studies on haematological malignancies have reported proteasome activity in the plasma to be a probable predictor of treatment response and survival [16, 17]. However, the influence of new drugs, mainly used in the treatment of multiple myeloma, on the proteasome parameters has not yet been subjected to evaluation.

Therefore, in this study, we set out to evaluate plasma proteasome concentration and ChT-L activity in healthy volunteers and MM patients in order to determine the usefulness of proteasomes as novel biomarkers which could facilitate the post-chemotherapy risk-group assessment and help to identify patients that will most likely respond to targeted agents. Furthermore, we investigated the association between proteasome concentration and ChT-L activity with response to targeted therapy and survival, with a view to determining the potential predictive and prognostic significance of the studied proteasome parameters.

## Materials

A total of 78 patients with newly diagnosed MM were included in this study. Their blood samples were available for collection right before the chemotherapy, after its third cycle and at the end of it, i.e. 4 weeks after the sixth cycle of chemotherapy. The median age of our patients at the time of sample collection was 67, and the range was 37–89. Patient samples were collected between the years 2014 and 2016 in the Department of Haematology at the Medical University of Bialystok, Poland. The diagnosis was conducted under the International Myeloma Working Group criteria [2]. In all the cases, we performed complete blood cells, total protein and albumin, protein immunoelectrophoresis, renal function tests, skeletal surveys and plasma cells in smear bone marrow. At the time of diagnosis, the patients were divided into three groups in accordance with the International Staging System (ISS): 16 patients in stage I, 25 patients in stage II and 37 patients in stage III. Patient characteristics are summarised in Table 1. MM therapy is tailored to each patient and depends on their age and physical condition. Patients below the age of 65 are eligible for the following chemotherapy types: CTD (cyclophosphamide, thalidomide, dexamethasone), VCD (bortezomib, cyclophosphamide, dexamethasone) or PAD (bortezomib, doxorubicin, dexamethasone). We recommended MPT (melphalan, prednisone, thalidomide) or VMP (bortezomib, melphalan, prednisone) for those who were not eligible for a transplant scheme. Twenty-six patients in our study were qualified for the CTD scheme of therapy and 52 patients for the therapy with proteasome inhibitor—bortezomib: 22 patients for the PAD therapy, 13 patients for the VCD therapy, 2 patients for the VTD therapy and 15 patients for the VMP therapy. After each therapy, we evaluated our patients’ response to treatment and divided them into the following categories: complete response (CR), very good partial response (VGPR), partial response (PR), stable disease (SD) and progressive disease (PD) [1]. Afterwards, the patients were divided into two groups: responders (CR + VGPR + PR), CTD 18 patients and PI 35 patients; and non-responders (SD + PD), CTD 8 patients and PI 17 patients. The response was assessed after the sixth cycle of chemotherapy based on the European, International, and Autologous Bone Marrow Transplant Registries (EBMT/IBMTR/ABMTR) criteria [18].

**Table 1:** Clinical features of the patients

	All MM patients	Patients treated CTD	Patients treated PI
Number of patients	*n* = 78	*n* = 26	*n* = 52
Age	67 (37–89)	68 (37–86)	66 (47–89)
Stage ISS			
I	*n* = 16	*n* = 4	*n* = 12
II	*n* = 25	*n* = 10	*n* = 15
III	*n* = 37	n = 12	n = 25
Solitary plasmocytoma	*n* = 0	n = 0	n = 0
HGB (g/dl)	10.45 (4.8–15.6)	10.4 (4.9–15.6)	10.55 (4.8–14.3)
Serum protein (g/dl)	8.2 (3.8–13.4)	8.55 (3.8–13.4)	7.9 (4.0–13.2)
Serum albumin (g/dl)	3.59 (1.9–5.0)	3.53 (1.9–5.0)	3.6 (2.05–4.2)
M protein (g/dl)	1.9 (0–5.6)	1.75 (0–5.6)	2.02 (0–4.5)
Ca^2+^ (mmol/l)	2.32 (1.53–3.99)	2.35 (1.81–3.95)	2.31 (1.53–3.99)
IgG (mg/dl)	2195 (153–11,100)	2892 (153–11,100)	2445 (163–9910)
β_2_M (g/l)	4.82 (1.86–43.02)	5.04 (2.32–16.01)	4.74 (1.86–43.02)
LDH (IU/l)	210 (115–693)	179.5 (115–314)	222.5 (117–693)
% plasma cell in smear BM	17 (0–87)	20 (0–71)	16 (0–87)
Creatinine level (mg/dl)	0.94 (0.53–6.77)	0.97 (0.53–6.15)	0.91 (0.61–6.77)
PLT (×10^3^)	194.5 (51–397)	222 (74–348)	192 (51–397)
Response to treatment (*n* (%))			
CR	8 (10%)	2 (8%)	6 (12%)
VGPR	20 (26%)	6 (23%)	14 (27%)
PR	25 (32%)	10 (38%)	15 (29%)
SD	7 (9%)	2 (8%)	5 (9%)
PD	18 (23%)	6 (23%)	12 (23%)

Response ≥VGPR: 36, 31, and 39% in the whole population, patients treated CTD, and patients treated PI, respectively; response ≥PR: 68, 69, and 68%, respectively. The values are presented as median (range)

*PI* proteasome inhibitor, *ISS* International Staging System, *HGB* haemoglobin, *M protein* monoclonal protein, *Ca*^*2+*^ calcium, *IgG* immunoglobulin G, *β*_*2*_*M* beta-2-microglobulin, *LDH* lactate dehydrogenase, *BM* bone marrow, *PLT* platelets counts, *CR* complete remission, *VGPR* very good partial response, *PR* partial response, *SD* stable disease, *PD* progressive disease

The study was approved by the Ethics Committee at the Medical University of Bialystok (Agreement No R-I-002/203/2014) and conducted in accordance with the 1964 Declaration of Helsinki and its later amendments. Informed consent was obtained from all the patients before including them in the study.

In the control group (age- and sex-matched), samples were obtained from 36 healthy volunteers.

## Methods

Peripheral venous blood was collected into the SARSTEDT blood-collection tubes containing a trisodium citrate (3.2%) anticoagulant and processed immediately by centrifugation at 1500*g* for 10 min at room temperature (+ 25 °C). Samples with no signs of haemolysis were stored at − 80 °C until analysis.

The ChT-L activity of circulating proteasomes was assessed through ongoing monitoring of the production of 7-amino-4-methylcoumarin (AMC) from fluorogenic peptide AMC substrate-Suc-Leu-Leu-Val-Tyr-7-amino-4-methylcoumarin (Sigma-Aldrich, USA) as presented by Ma et al. [16, 17]. First of all, plasma samples were activated with 5 μl 10% SDS at room temperature for 15 min. The reaction wells contained 30 μl assay buffer (0.05% SDS in 100 mM Tris/HCL, pH 7.5), 10 μl activated plasma and 10 μl fluorogenic peptide-AMC substrate. Volume of the release of free AMC with time was measured by FLUOstar OPTIMA (BMG Labtech, Germany) with the following parameters: excitation of 355 nm, emission of 460 nm and read length of 60 min at 37 °C. The amount of AMC released per minute (pmol/min = U) was established as a single unit of the 20S proteasome ChT-L activity. This activity was calculated for the amount of total protein (U/mg). The concentration of total proteins in our plasma samples was determined by the Bradford method in Biofotometr (Eppendorf), using the Bio-Rad assay reagent [19].

The concentration of circulating proteasome in our plasma samples was measured by means of a commercial ELISA kit (Enzo Life Sciences, USA).

## Statistics

The results are presented as medians and range. Associations between our MM patients and control groups were evaluated for continuous parameters using the Mann–Whitney *U* test. For the longitudinal comparison of pre-treatment versus after the third cycle of chemotherapy versus post-treatment samples, the Wilcoxon test was applied. Survival curves were created with the Kaplan–Meier method, and the log-rank test was used to determine differences between survival proportions. Cox proportional hazards model was applied to assess the prognostic strength of proteasome ChT-L activity and concentrations. PFS was defined as the amount of time between treatment introduction and any event, such as disease progression, death from any cause or the last date on which the disease activity was evaluated.

For all the tests, *p* values less than 0.05 were considered as statistically significant.

## Results

### Proteasome parameters in healthy donors and newly diagnosed MM

MM patients displayed a significantly higher proteasome ChT-L activity and proteasome concentration in the plasma compared to healthy volunteers (activity: median 1.26 U/mg, range 0.22–3.55 vs 0.79 U/mg; 0.21–1.72 U/mg, *p* = 0.0068; concentration: median 2.11 μg/ml, range 0.54–7.33 vs 1.36 μg/ml; 0.43–3.71 μg/ml, *p* = 0.002). Patients in stage I MM and patients in stages II and III (following the ISS) were analysed separately and showed an increase, although not significant, in the studied parameters according to the degree of disease stage advancement (data not shown), for activity, *p* = 0.65, and concentration, *p* = 0.76, respectively.

There was no meaningful difference in proteasome concentration and ChT-L activity between the IgG and IgA MM patients (data not shown).

### Proteasome parameters throughout treatment in the CTD group and PI-based group

The subgroups, i.e. groups of patients treated either with CTD or PI, did not show any meaningful differences with regard to ISS, sex, age or creatinine level (for all, *p* > 0.05).

In the group of patients treated with PI, plasma proteasome ChT-L activity and concentration decreased significantly at the end of treatment compared to their baseline values (*p* = 0.004, *p* = 0.001, respectively, Table 2). On the other hand, in the CTD group of patients, the data showed no significant changes between the baseline proteasome ChT-L activity and its value at the end of chemotherapy (*p* = 0.69). The increase from the baseline value of proteasome concentration to the level at the end of chemotherapy was insignificant (*p* = 0.32, Table 2).

**Table 2:** The median values of proteasome ChT-L activity and proteasome concentration in plasma of patients with MM treated with bortezomib or CTD

No. of patients		Parameters					
		Proteasome ChT-L activity (U/mg)			Proteasome concentration (μg/ml)		
		Baseline	After third cycle	End of treatment	Baseline	After third cycle	End of treatment
Bortezomib	All patients (*n* = 52)	1.24 (0.22–3.55)	0.93* (0.27–5.68)	0.87** (0.22-3.65)	2.48 (0.65–11.79)	2.12 (0.53–7.15)	1.79** (0.47–4.26)
	Responders (*n* = 35)	1.52 (0.35–3.55)	0.81* (0.27–2.08)	0.69** (0.22–1.73)	2.81 (0.79–11.79)	2.05* (0.53–5.3)	1.6** (0.47–3.9)
	Non-responders (*n* = 17)	0.66 (0.22–1.79)	1.19 (0.29–5.68)	1.24** (0.49–3.65)	1.79 (0.65–4.29)	2.27 (0.58–7.15)	2.19 (0.92–4.26
CTD	All patients (*n* = 36)	1.15 (0.23–1.73)	0.82 (0.32–1.81)	0.88 (0.27–4.03)	1.72 (0.54–5.05)	1.99 (0.7–4.74)	2.01 (0.79–7.31)
	Responders (*n* = 18)	0.99 (0.36–1.73)	0.78* (0.32–1.81)	0.99** (0.27–4.03)	1.93 (0.62–5.05)	2.09 (0.9–4.74)	1.83 (0.79–3.15)
	Non-responders (*n* = 8)	0.62 (0.23–1.42)	0.7 (0.38–0.98)	0.88 (0.53–2.03	1.25 (0.54–2.64)	1.77 (0.7–3.6)	2.04 (1–7.31)

The values are presented as median (range). Responders are patients, who received at least partial response; non-responders are patients with stable and progressive disease

*ChT-L* chymotrypsin-like, *CTD* cyclophosphamide, thalidomide, dexamethasone

*p < 0.05 between baseline value and after third cycle

**p < 0.05 between baseline value and end of treatment

### Proteasome parameters throughout the treatment in the study subgroups, including clinical response

**Fig. 1:**
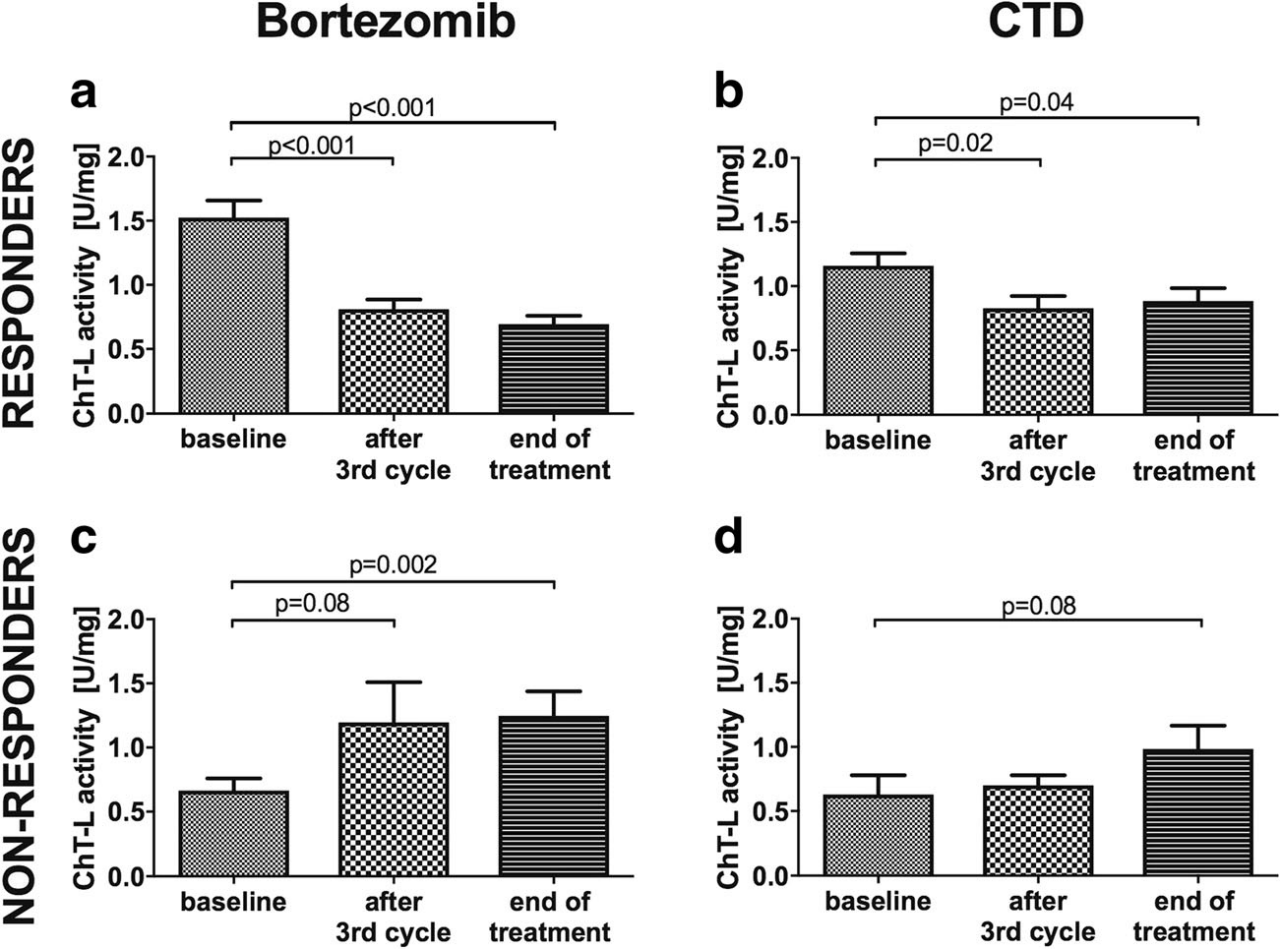
The proteasome chymotrypsin-like (ChT-L) activity in multiple myeloma patients treated with bortezomib or CTD. **a** Responders patients treated with bortezomib. **c** Non-responders patients treated with bortezomib. **b** Responders patients treated with CTD. **d** Non-responders patients treated with CTD. ChT-L chymotrypsin-like activity, CTD cyclophosphamide, thalidomide, dexamethasone. Responders are patients, who received at least partial response; non-responders are patients with stable and progressive disease

**Fig. 2:**
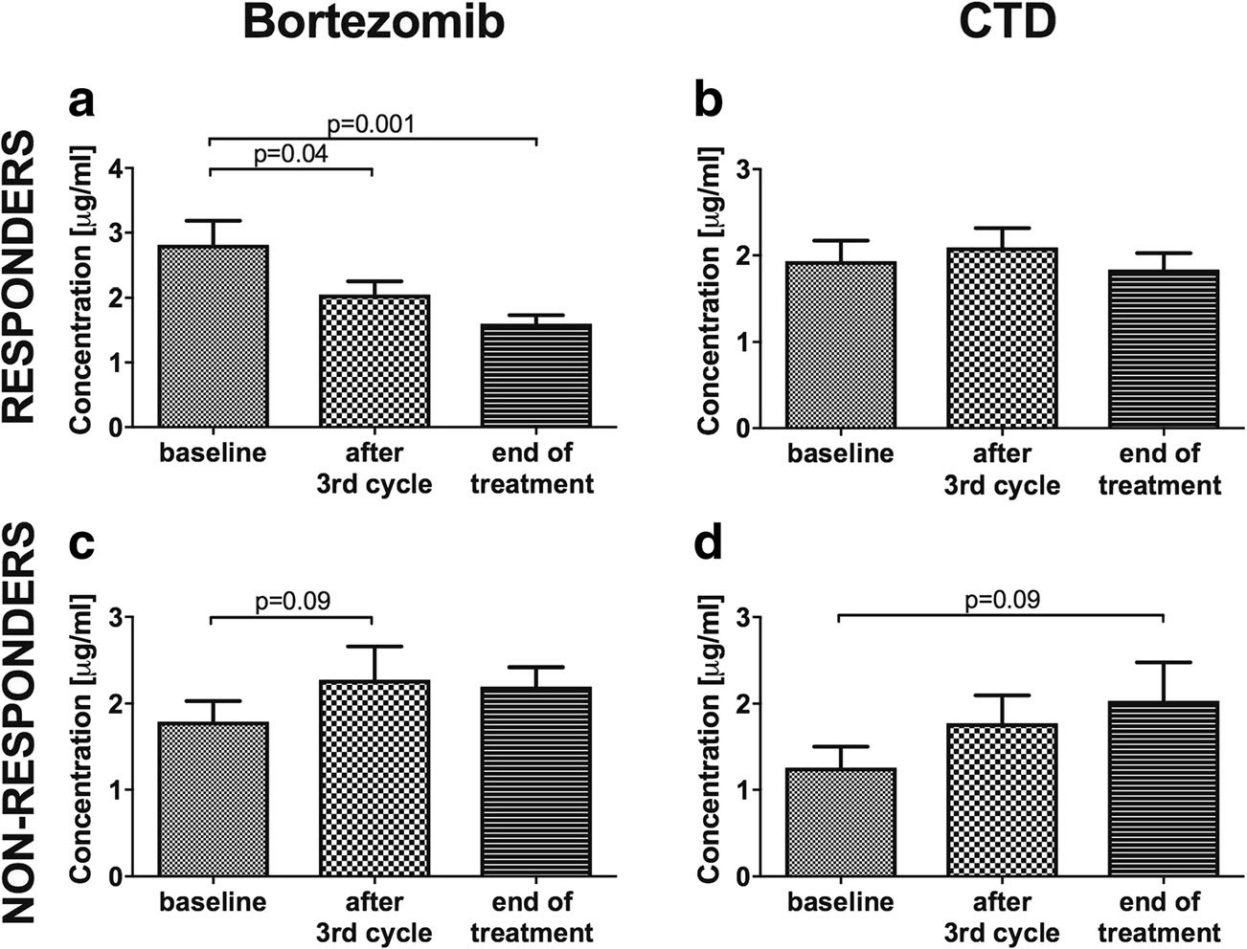
The proteasome concentration in multiple myeloma patients treated with bortezomib or CTD. **a** Responders patients treated with bortezomib. **c** Non-responders patients treated with bortezomib. **b** Responders MM patients treated with CTD. **d** Non-responders MM patients treated with CTD. CTD cyclophosphamide, thalidomide, dexamethasone. Responders are patients, who received at least partial response; non-responders are patients with stable and progressive disease

First of all, in the group of patients treated with PI, and more precisely those who achieved at least PR, the values of proteasome ChT-L activity and proteasome concentration at the third cycle and at the end of chemotherapy were significantly lower than the baseline (Figs. 1 and 2a and Table 2). Median ChT-L activity levels in the group of PI patients after the third cycle of treatment were reduced by about 50% (*p* < 0.001) and remained low at the end of therapy (*p* < 0.001). This meaningful decrease in the activity but not concentration was shown as well in our CTD responders (Figs. 1 and 2b and Table 2). The reduction in the latter group was much lower, amounting to merely one third (*p* = 0.02 after third cycle and *p* = 0.04 at the end of therapy, respectively). The study did not reveal a change in concentration in the CTD responders (*p* = 0.5).

Even more importantly, in the group of patients treated with PI who did not achieve remission (patients with SD and PD) after the chemotherapy, the study showed an opposite correspondence. In this group, we demonstrated that proteasome ChT-L activity and concentration were enhanced during the time of treatment. Median ChT-L activity levels after the third cycle of treatment were elevated by about 22% (*p* = 0.08) compared to their baseline values and increased after the treatment by about 60% (*p* = 0.002) (Fig. 1c, Table 2). Proteasome concentration, however, rose only slightly (*p* = 0.09) (Fig. 2c, Table 2). In the control group (CTD non-responders), the analysis showed only a modest increase in the activity and concentration at the end of therapy compared to their baseline values, standing at the limit of significance (*p* = 0.06 for both respectively) (Figs. 1 and 2d and Table 2).

### Assessment of the prognostic value of proteasome parameters

In order to assess the prognostic value of the studied parameters, we set out to analyse the effect of baseline proteasome ChT-L activity and concentration on the type of response according to the type of chemotherapy (PI vs CTD). Statistically significant differences were found between the baseline proteasome ChT-L activity in the group of patients who achieved at least partial response and patients who did not respond to treatment regardless of the used scheme of chemotherapy (PI: median 1.69 U/mg, range 0.82–3.55 vs 0.66 U/mg; 0.22–1.79 U/mg, *p* < 0.0001) (CTD: median 1.17 U/mg, range 0.36–17.73 vs 0.65 U/mg; 0.23–1.42 U/mg, *p* = 0.01). The study established that pre-treatment values of proteasome ChT-L activity predicted better response to chemotherapy, as the patients who achieved at least PR had a significantly higher baseline value compared to non-responders. On the other hand, there were no significant changes in the baseline value of proteasome concentration between responders and non-responders regardless of the applied scheme of therapy (PI: median 2.32 μg/ml, range 0.91–11.79 vs 1.87 μg/ml; 0.65–4.29 μg/ml, *p* = 0.1) (CTD: median 1.94 μg/ml, range 0.62–5.05 vs 1.25 μg/ml; 0.54–2.64 μg/ml, *p* = 0.09).

### Association between proteasome parameters and clinical outcomes

We subsequently correlated proteasome concentration and ChT-L activity with a selection of well-known parameters of prognosis and tumour load in MM listed in Table 1. Plasma proteasome concentration correlated significantly and positively with β_2_-M level (rho = 0.49, *p* = 0.0007, data not shown) and creatinine level (rho = 0.32, *p* = 0.01, data not shown). Plasma proteasome ChT-L activity correlated significantly and positively with LDH activity (rho = 0.42, *p* = 0.0019, data not shown) and with β_2_-M concentration at the limit of statistical significance (rho = 0.23, *p* = 0.09, data not shown). We observed the existence of a negative correlation standing at the limit of significance between the level of immunoglobulins and a low value of proteasome ChT-L activity particularly in the group of non-responders (rho = − 0.25, *p* = 0.09, data not shown).

More importantly, the study showed a positive and significant correlation between the baseline proteasome ChT-L activity and proteasome concentration (*p* < 0.0001).

**Fig. 3:**
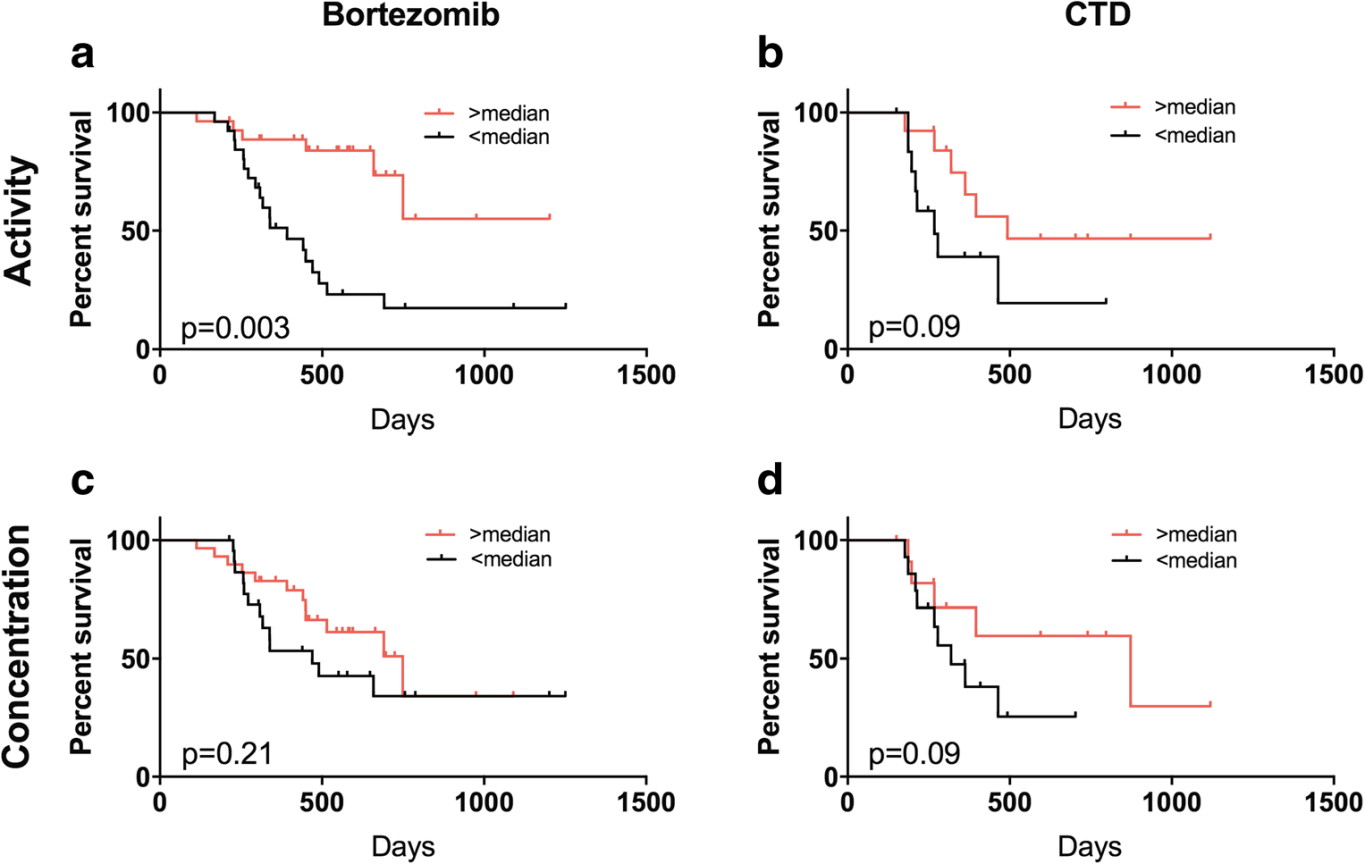
Kaplan–Meier curves of progression-free survival estimates according to plasma proteasome chymotrypsin-like activity in multiple myeloma patients treated bortezomib (**a**) or CTD (**b**), and plasma proteasome concentration in multiple myeloma patients treated bortezomib (**c**) or CTD (**d**). CTD cyclophosphamide, thalidomide, dexamethasone

Additionally, we observed that patients with a pre-treatment value of proteasome ChT-L activity higher than the median had a significantly longer PFS compared to subjects with lower than the median proteasome ChT-L activity, depending on the used scheme of chemotherapy (PI: *p* = 0.0003, CTD: *p* = 0.09) (Fig. 3a, b). On the other hand, there were no statistically significant differences with reference to PFS in the groups of MM patients with regard to the median values of concentration, regardless of the schema *p* = 0.21 and *p* = 0.09 (Fig. 3c, d). Multivariate Cox proportional hazard model, which incorporates all significant factors along with the studied protein, showed that only proteasome ChT-L activity but not concentrations can be considered as risk factor independent of age (< 65 or ≥ 65), ISS, type of regiment or gender.

## Discussion

Dysregulation of the ubiquitin-proteasome system (UPS) leads to uncontrolled cell proliferation and tumour growth. The recent studies have established the assessment of proteasome concentration and ChT-L activity to constitute a new approach to diagnosis, prognosis and monitoring of treatment in patients with haematological malignancies [6, 7, 10, 20], while access to biomarkers predictive of clinical responses could help clinicians tailor the treatment for myeloma patients. Unfortunately, the influence of new drugs, used predominantly as part of regiments to treat MM, on proteasome parameters has not yet been subjected to evaluation.

The present study is the first one to have analysed simultaneously plasma concentration and ChT-L activity of proteasomes in a cohort of patients with newly diagnosed MM before, during and after treatment. First of all, the study confirmed some significantly greater differences in the study proteasome parameters of MM patients compared to healthy volunteers, which are consistent with those obtained by others, including a studies concerning patients with solid tumours and haematological malignancies [6, 7, 10–12, 20]. Unfortunately, the source of an elevated proteasome level remains unclear. It has been suspected that proteasome levels originate from both tumour cells and non-malignant cells as a result of an immune reaction [21]. On the other hand, the proteasome concentration in our study was weakly correlated with laboratory parameters of prognosis and tumour load in patients with MM. We were also noted higher levels of proteasome ChT-L activity in ISS stage III compared to stage I, although it did not acquire statistical significance. We observed a similar situation in proteasome concentration as Manasanch et al. [22], who examined proteasome activity in newly diagnosed MM patients treated with carfilzomib, lenalidomide and dexamethasone: There were no statistically significant differences with regard to the ISS stage. On the contrary, data from another study showed meaningfully increased ChT-L activity and proteasome concentration in a group of patients in advanced stages of MM [7, 12] and other diseases, such as CLL patients [16], and with solid tumours in stages III/IV [20]. On the other hand, the study showed ChT-L activity to correlate with prognostic factors, such as LDH [6, 7, 17], and with β2-M concentration, which reflects the activity of MM and may be related to the growth of myeloma cells [17].

It is worth noticing that studies of proteasome parameters in patients before, during and after chemotherapy are currently rare. Our analysis shows that values of all the studied parameters (activity and concentration) decreased sharply from their initial levels already after the third cycle of chemotherapy in the group of patients who responded to therapy and remained significantly lower until the end of treatment, which was evident mainly in patients treated with PIs. The results obtained in our study revealed no significant differences between proteasome parameters dependent on the kind of treatment, be it CTD or PI. The decrease in the CTD group can be explained by the use of corticosteroid, an important part of treatment regiments in MM, or the effect of thalidomide or cyclophosphamide on the activity of proteasomes. Indeed, the research by Beyette et al. showed a decrease in proteasome ChT-L activity following treatment with dexamethasone in thymocytes [23].

Unfortunately, the mechanism of proteasome elimination from the organism remains unknown. Ostrowska et al. [6] proved reduced levels of ChT-L activity of 20S proteasome in AML and ALL patients after complete remission and unchanged or increased levels in patients showing no remission, which suggests that proteasomes are likely to be cleared after the elimination of malignant tumour cells. Moreover, the above-mentioned study demonstrated that changes in plasma 20S proteasome ChT-L activity during cytotoxic treatment, remission and relapse correlated with changes in the serum LDH activity [10, 11].

In our study, we also noted an increase in the measured proteasome parameters in the non-responders group of patients, which was evident already after the third cycle of chemotherapy, prior to being detected on the basis of tests such as immunofixation of disease progression, and it remained high until the end of treatment (disease stabilisation or proofed progression). Similar results were demonstrated by Jakob et al. that proteasome concentration was significantly elevated during active disease (non-responders) and decreased significantly in the post-therapy samples of the responders [12]. Thus, the association between the baseline proteasome ChT-L activity and clinical response suggests that plasma proteasome ChT-L activity might be reflective of the activity inside malignant plasma cells.

The most important observation stemming from our analyses is the fact that patients who did not respond to the applied chemotherapy had a statistically significant lower level of proteasome ChT-L activity compared to responders. This phenomenon was most clearly visible in the group of patients treated with PI and might be due to the mutations of the β5 binding pocket of the proteasome, resulting in conformational changes to the bortezomib-binding pocket of this subunit and resistance to this drug [24]. The method of evaluating proteasome ChT-L activity used in our study was not likely to detect mutated proteasomes activity. Therefore, further studies are necessary to address these aspects.

It is also worth emphasising that Meister et al. discovered in one of their studies that proteasome inhibitors induce apoptosis preferentially in the cells with a high synthesis rate of immunoglobulin, associated with the accumulation of unfolded proteins/DRiPs inducing ER stress [25]. Similarly, we observed the existence of a negative correlation standing at the limit of significance between the level of immunoglobulins and a low value of proteasome activity particularly in the group of non-responders.

Last but not least, our study revealed meaningful differences between the initial proteasome activity and concentration in MM patients prior to treatment commencement. To our surprise, subjects with higher initial activity achieved complete remission far more often. The group of patients treated with PI and CTD did likewise. Nevertheless, our findings suggest that proteasome ChT-L activity may constitute a marker for predicting response to standard chemotherapy independent of the kind of implemented treatment. Predictive markers are factors that help clinicians personalise therapies for their patients. Identification of this factor maximises good outcomes and minimises side effects, as well as treatment burden.

It is similarly crucial to predict event-free survival. Our study showed that MM patients with baseline proteasome ChT-L activity values higher than the median of all the MM patients had a significantly longer survival than patients with a lower proteasome value. These meaningful differences in PFS were observed to a greater extent in the PI group than in the CTD. The data presented here show that ChT-L activity is a strong predictor of survival, dependent on the administered treatment.

In conclusion, the data presented in our study show that ChT-L activity might be used to predict event-free survival, but prospective clinical trials are required to evaluate the usefulness of this approach in guiding therapy decisions. Our findings demonstrate that measuring plasma proteasome ChT-L activity can be employed as a powerful biomarker for predicting clinical response to treatment and survival in patients with newly diagnosed MM. This will allow us to identify relatively early groups of patients with a high probability of responding to chemotherapy versus those who will not prior to the introduction of treatment.
